# Clinicopathologic and microenvironmental analysis of primary cutaneous CD30-positive lymphoproliferative disorders: a 26 year experience from an academic medical center in Brazil

**DOI:** 10.1186/s13000-019-0900-7

**Published:** 2019-10-22

**Authors:** Cristiane Rúbia Ferreira, Shuchun Zhao, José Antonio Sanches, Denis Miyashiro, Jade Cury-Martins, Raymundo Soares Azevedo, Maria C. N. Zerbini, Yasodha Natkunam, Dita Gratzinger

**Affiliations:** 1 Department of Pathology, Stanford University School of Medicine, Stanford, CA 94305 USA; 2 Departments of Pathology, University of Sao Paulo, Sao Paulo, SP 01246 Brazil; 3 Anatomical Pathology Service, University Hospital of Sao Paulo University (HU-USP), Rua Professor Lineu Prestes, 2565, Sao Paulo, SP 05508-000 Brazil; 4 Departments of Dermatology, University of Sao Paulo, Sao Paulo, SP 01246 Brazil

**Keywords:** Primary cutaneous CD30+ T-cell c, Cutaneous lymphoma, FOXP3, Regulatory T-cell, CD8, Programmed death ligand 1, Tumor microenvironment, Immunohistochemistry

## Abstract

**Background:**

Primary cutaneous CD30+ lymphoproliferative disorders (pc-CD30-LPD) are a group of clonal T cell lymphoproliferative disorders that despite very similar tumor histology follow different and characteristic clinical courses, suggesting a homeostatic role of the tumor microenvironment. Little is known about tumor microenvironment and there is almost no literature about PD-L1 expression in pc-CD30-LPD.

**Methods:**

This retrospective study presents a fully clinicopathologically characterized series of pc-CD30-LPDs from an academic medical center in Brazil, including 8 lymphomatoid papulomatosis (LyP), 9 primary cutaneous anaplastic large cell lymphoma (pcALCL) and 4 borderline lesions. All the cases were scored for FOXP3+ regulatory T-cells (Treg) and CD8+ cytotoxic tumor infiltrating lymphocytes (TIL) densities, as well as PD-L1 expression in tumor cells and tissue associated macrophages. The CD8+/FOXP3+ ratio was also evaluated.

**Results:**

Among the 21 cases of pc-CD30-LPD, PD-L1 expression is frequent in both tumor cells and tissue associated macrophages in pc-CD30-LPD across categories, suggesting that the PD-L1 axis may be a common feature of pc-CD30-LPDs. While reactive T cell infiltrates vary widely from case to case, a common feature across pc-CD30-LPDs is higher density of CD8 than FOXP3 + T cells. The distribution of T cells within the lesions however differed between LyP and pcALCL: we found that LyP lesions tend to be permeated by CD8+ and FOXP3+ T cells, whereas pcALCL tend to be surrounded by a rim of CD8+ TIL and FOXP3+ Tregs with relatively lower density infiltrates in the center of the lesion.

**Conclusions:**

LyP has a trend to have denser immune cells throughout the lesion, with higher FOXP3+ Treg and CD8+ TIL in the center than the edge comparing with pcALCL. PD-L1+ is frequent in tumor cells and tissue associated macrophages in pc-CD30-LPD. The differential distribution of CD8+ and FOXP3+ TILs in LyP as compared to pcALCL could provide a clue to the relapsing/remitting course of LyP as compared to the less frequent spontaneous regression of pcALCL.

## Introduction

Primary cutaneous CD30+ lymphoproliferative disorders (pc-CD30-LPD) include the closely related T lymphoproliferative disorders primary cutaneous anaplastic large cell lymphoma (pcALCL), lymphomatoid papulosis (LyP) and borderline lesions [1]. Classification requires clinicopathologic correlation by an experienced dermatologist and pathologist. pcALCL has a good prognosis when compared to systemic ALCL [1, 2], even in the presence of regional lymphadenopathy, and may regress spontaneously [3, 4]. LyP has a characteristic self-resolving, recurring clinical course. Borderline lesions are clinicopathologically intermediate. The typical waxing and waning course of LyP, and the indolent course and occasional spontaneous regression of pcALCL, raise the question of the role of the tumor microenvironment in the clinical course of pc-CD30-LPDs.

Immune escape is an active process of immune evasion by neoplastic cells. Malignant cells may suppress host immunity directly by secreting immunoregulatory cytokines, by recruiting immunoregulatory cells capable of suppressing host immunity, or by over-expressing the ligands of checkpoint receptors on their surface and bringing T cells to a state of intrinsic dysfunction that leads to an anergic or exhaustion state [5–7]. Thus, the density of regulatory T-cells (Treg), CD8+ cytotoxic tumor infiltrating lymphocytes (TILs), and the expression of programmed death ligand 1 (PD-L1) by the tumor tissue, represent multiple interfaces contributing to the homeostasis of the tumor microenvironment [6–8]. PD-L1 is a co-inhibitory ligand that hampers the effector phase of the immune response, such as by inducing and sustaining Treg cell function [9–11]. Treg are a subset of CD4+ helper T-cells which induce functional exhaustion in CD8+ T-cells [11]. FOXP3, a forkhead helix transcription factor, is considered the most specific and reliable marker for Treg [12]. Little is known about the tumor microenvironment and there is almost no literature about PD-L1 expression in pc-CD30-LPDs [13–15]. We provide here a study of the expression of PD-L1 and the density of FOXP3 Treg and CD8+ T-cells to better understand the role of tumor microenvironment in a well characterized cohort of pc-CD30-LPD from an academic medical center in Brazil.

## Material and methods

### Tissue samples

The study cohort included 26 cases of pc-CD30-LPD which were diagnosed during the period from 1990 through 2016 from the archives of the Dermatopathology Laboratory, Department of Dermatology of Clinics Hospital/ Sao Paulo University Faculty of Medicine (HC/FMUSP) from Brazil. All patients were seen and staged [16] by a clinical dermatologist with experience in cutaneous lymphoid disorders. All the slides were reviewed by two pathologists (CRF and DG). The clinical and pathological definition of pc-CD30-LPD used in this study is consistent with that of the World Health Organization – European Organization for Research and Treatment of Cancer (WHO – EORTC) classification for cutaneous lymphomas and the 4th revised edition of WHO classification, 2017 [1, 2]. A total of 21 patients were enrolled in our study: 8 with LyP, 9 with pcALCL and 4 with borderline lesions. A total of 5 patients were excluded; of these, 3 cases had scant tissue, 1 case had inflammatory cells but did not contain CD30+ tumor cells in the remaining tissue, and 1 case was a systemic ALK-negative ALCL with secondary skin involvement. The research was given official approval by the local Ethical Committee (CAPPesq n° 15,486).

### Immunohistochemistry

Immunohistochemistry was performed on a standardized automated staining system, Ventana Benchmark XT (Retrieval: Tris/ Borate/ EDTA buffer, pH 8.0–8.5) for ALK (clone: ALK1; Dako) and EBV ISH - in situ hybridization for EBV-associated small RNAs (EBER) (Retrieval: protease), Leica BOND-III (Leica Epitope Retrieval 2: Tris-EDTA buffer, pH 9.0) for ALK (clone: 5A4; Abcam), CD30 (clone: Ber-H2; Dako), CD8 (clone: C8/144B; Dako), FOXP3 (clone: 236A/E7; Abcam) and D2–40 (clone: D2–40; Dako: no retrieval), and manual pressure cooker instrument (Retrieval: EDTA (1 mM)/Tris (5 mM) at pH 9 for 10 min) for PD-L1 (clone: E1L3N; Cell Signaling), CD3 (polyclonal; Dako) and CD20 (clone: L26; Dako).

PD-L1 expression in cytoplasm and/or membrane was considered positive; PD-L1 expression of tumor cells and of non-tumor infiltrating cells histologically compatible with tissue associated macrophages (TAM) was scored separately. The scoring schema for PD-L1 in each cell type was: Negative, less than 5% positivity; Weak, ≥5 to < 30% positivity or very weak intensity; Strong, ≥30% positivity with moderate to strong intensity.

Quantitative evaluation of CD8+ and FOXP3+ Treg TILs were performed by examining 2 non-overlapping high-power fields (HPF – 40X objective) in the center of the tumor and 2 non-overlapping HPF at the edge/border of the tumor in each stained slide. The mean numbers of CD8+ TILs and FOXP3+ Treg TILs were calculated for the center and edge respectively. The CD8+/FOXP3 Treg ratio for the center and the edge was defined as the mean number of CD8+ TILs divided by the mean number of FOXP3 Treg TILs per field in each case.

### Statistical analysis

Immunohistochemical variables are classified according to the intensity observed in the tissue, therefore being possible to ordinate the results. These variables are also not expected to have any particular distribution, being more appropriate to use distribution free statistical methods to analyze the collected data. Therefore, in order to compare the outcomes from the three groups, the non parametric statistical methods used were the Kruskall-Wallis test, Wilcoxon signed-rank test, Fisher’s Exact Test and Spearman’s rank correlation coefficient. The level of significance is 5% (=0.05), using Bonferroni correction for multiple comparisons when Kruskall-Wallis test shows statistical difference among the three groups of patients for a given variable. Statistical analysis was performed using IBM SPSS version 23.0 (Statistical Package for Social Sciences). Survival analysis for time to first relapse was performed with Cox proportional hazards regression on Stata/SE 15.1 for Mac (Stata Corp, College Station, TX).

## Results

### Clinicopathological features

Clinicopathologic features are summarized in Table 1. All patients with LyP (7 LyP type A and 1 LyP type C) had characteristic self-healing lesions, symptom-free period(s) and recurrence. One patient with LyP had a subsequent diagnosis of mycosis fungoides (MF) 19 years later. The clinical presentation of the pcALCL lesions was described as erythematous or infiltrated plaques, ulcerated nodules or tumors. One patient with pcALCL had a subsequent diagnosis of MF 2 years later. The clinical presentation of patients with borderline lesions was described as erythematous, infiltrated plaques and papules, with associated ulcerated tumor. One patient with a borderline lesion had a histological diagnosis of pcALCL in a biopsy of a solitary nodule on the face, but by clinic-dermatological evaluation she also presented generalized self-healing papules and nodules characteristic of LyP, so a diagnosis of borderline lesion was rendered by dermatology-pathology correlation. There was no statistically significant difference in age, length of followup, or number of recurrences among the 3 groups (Kruskall-Wallis test). Nineteen of the total group of 21 patients were alive at the end of follow up; 4 of 21 were in remission and the only two patients who had died had causes of death not related to their cutaneous T cell lymphoproliferative disorders. None of the LyP patients showed regional lymph node involvement. Due to the small numbers and poorly defined nature of the borderline group, only confirmed pcALCL and LyP cases were included in analyses comparing diagnostic subtypes of pc CD30+ ALCL.

**Table 1:** Clinicopathological data

Clinicopathologic Diagnosis	Histologic Diagnosis	Gender	Age (years)	Follow-up (months)	Relapses (n)	First line treatment	Stage
All patients Median (std dev)		11:10 M:F	53 (14)	63 (90)	3 (2)		
pcALCL*	pcALCL	M	48	43	1	Radiotherapy, CHOP, bortezomib	T3bN0M0
pcALCL	pcALCL	M	33	22	1	Radiotherapy	T3bN0M0
pcALCL	pcALCL	M	69	92	3	Radiotherapy	T1bN1M0
pcALCL	pcALCL	F	73	26	N/A	N/A	T1aN1M1
pcALCL	pcALCL	M	32	117	3	ProMACE CytaBOM	T3bN0M0
pcALCL	pcALCL	F	58	4	0	N/A	T1aN0M0
pcALCL	pcALCL	M	46	7	0	Radiotherapy	T1bN0M0
pcALCL	pcALCL	F	46	64	5	Excision	T2aN0M0
pcALCL	pcALCL	M	83	17	0	None	T2aN1M0
pcALCL - Median (std dev)		6:3 M:F	52 (17)	24 (37)	1 (2)		
Borderline	pcALCL	F	53	293	7	Excision	T1aN0M0
Borderline	Borderline	F	58	110	3	None	T3aN0M0
Borderline	Borderline	F	64	63	N/A	Excision	T3bN0M0
Borderline	Borderline	F	49	3	0	Prednisone	T3bN0M0
Borderline - Median (std dev)		0:4 M:F	56 (6)	87 (108)	3 (3)		
LyP	LyP-C	F	34	214	5	Topical corticosteroid	
LyP*	LyP-A	F	47	334	4	Prednisone, cyclophosphamide	
LyP	LyP-A	M	57	86	N/A	N/A	
LyP	LyP-A	F	29	118	4	Phototherapy^	
LyP	LyP-A	M	54	16	1	Methotrexate	
LyP	LyP-A	M	43	87	5	Phototherapy	
LyP	LyP-A	M	61	11	N/A	Methotrexate	
LyP	LyP-A	M	59	40	1	Topical corticosteroid	
LyP - Median (std dev)		5:3 M:F	51 (11)	87 (103)	4 (2)		

*CHOP* Cyclophosphamide, hydroxydaunorubicin, oncovin, prednisone; *LyP* Lymphomatoid papulomatosis, *LyP-A* Lymphomatoid papulomatosis type A, *LyP-C* Lymphomatoid papulomatosis type C, *N/A* Not available., *pcALCL* primary cutaneous anaplastic large cell lymphoma, *ProMACE CytaBOM* Cyclophosphamide, hydroxydaunorubicin, etoposide, cytarabine, bleomycin, oncovin, methotrexate and prednisone, *std. dev* standard deviation

*Subsequent diagnosis of mycosis fungoides; ^Phototherapy = psoralen combined UVA

The atypical lymphoid cell component was CD30-positive, ALK-negative and EBER-negative in all cases. Occult intralymphatic involvement was assessed by staining with D2–40. Definitive lymphoma cells within lymphatic vessels were found in 10 of 20 evaluable cases. There was no statistically significant association between the presence and absence of a demonstrated intralymphatic component and histological subtype, age, sex, stage, regional nodal disease, relapse or clinical status at the last follow-up (data not shown).

#### Quantitation of FOXP3+ regulatory T cell and CD8+ T cell infiltrates

##### FOXP3-positive regulatory T-cells are preferentially enriched in the center of LyP as compared to pcALCL lesions

**Fig. 1:**
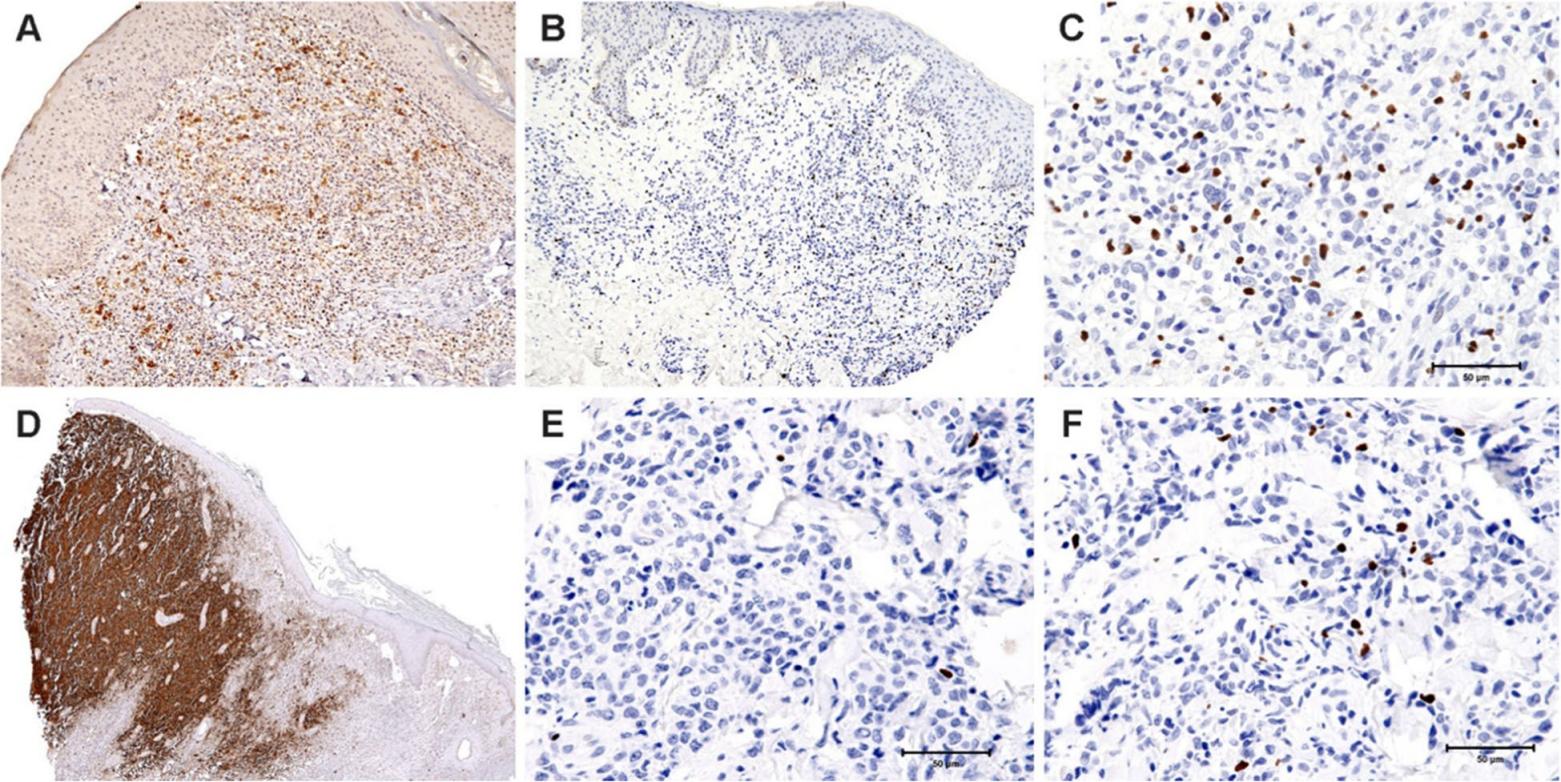
FOXP3 density in lymphomatoid papulomatosis (LyP) and primary cutaneous anaplastic large cell lymphoma (pcALCL): **a**- LyP showing few CD30 positive neoplastic cells (100X). **b**- FOXP3 positive cells distribution throughout LyP lesion (100X). **c**- The distribution of FOXP3 positive cells was enriched in the center of LyP (400X). **d**- pcALCL showing diffuse positivity for CD30 (100X). **e**- Presence of scattered FOXP3 positive cells in the center of pcALCL (400X). **f**- The distribution of FOXP3 positive cells was enriched in the edge of pcALCL (400X)

We assessed both the density of FOXP3+ regulatory T cells at both the edges of the neoplastic large cell aggregates and in the center of the lesions (Fig. 1). There was a trend toward a denser FOXP3+ T cell infiltrate in the center but not the edge of LyP cases as compared to pcALCL cases (*p* = 0.05, Kruskall-Wallis test, Table 2). Indeed, LyP but not pcALCL lesions showed significantly higher density of FOXP3-positive cells in the center of the lesions as compared to the edge when a paired test comparing the center and edge of each lesion was performed (*p* = 0.0499; Wilcoxon signed-ranks test). There was also a statistical trend toward a difference in the ratio of edge vs center FOXP3+ cells between LyP (0.9 edge to center ratio) and pcALCL (3.6 edge to center ratio). The difference in ratios between the two groups approached statistical significance (*p* = 0.05, Kruskall-Wallis test, Table 2). The absolute density of FOXP3+ regulatory T cell cuffs around the edges of the lesions on the other hand were not significantly different between LyP and pcALCL groups (*p* = 0.61; Kruskal-Wallis test; Table 2).

**Table 2:** Tumor microenvironment analysis: Comparing average FOXP3 Treg and CD8 TIL density at the center or edge of tumor lesions

Tumor Microenvironment	Diagnosis (n)	Mean (Standard deviation)	*LyP vs pcALCL
Tumor center CD8 TIL density (per hpf)	LyP (8)	87 (69)	*p* = 0.14
	pcALCL (8)	47 (39)	
Tumor center FOXP3 Tregs density (per hpf)	LyP (8)	56 (41)	*p* = 0.05
	pcALCL (9)	23 (27)	
Tumor edge CD8 TIL density (per hpf)	LyP (8)	67 (18)	*p* = 0.05
	pcALCL (6)	130 (79)	
Tumor edge FOXP3 Treg density (per hpf)	LyP (8)	31 (23)	*p* = 0.61
	pc-ALCL (6)	36 (21)	
Ratio CD8 TIL edge/center	LyP (8)	1.0 (0.5)	*p* = 0.04
	pcALCL (6)	2.6 (1.9)	
Ratio FOXP3 Treg edge/center	LyP (8)	0.9 (0.9)	*p* = 0.05
	pcALCL (6)	3.6 (3.6)	

*Kruskal-Wallis test; *LyP* Lymphomatoid papulomatosis, *pcALCL* primary cutaneous anaplastic large cell lymphoma, *TILs* tumor infiltrating lymphocytes, *HPF* High-power fields

##### CD8-positive tumor infiltrating lymphocytes outnumber are preferentially enriched at the edge of pcALCL as compared to LyP lesions

**Fig. 2:**
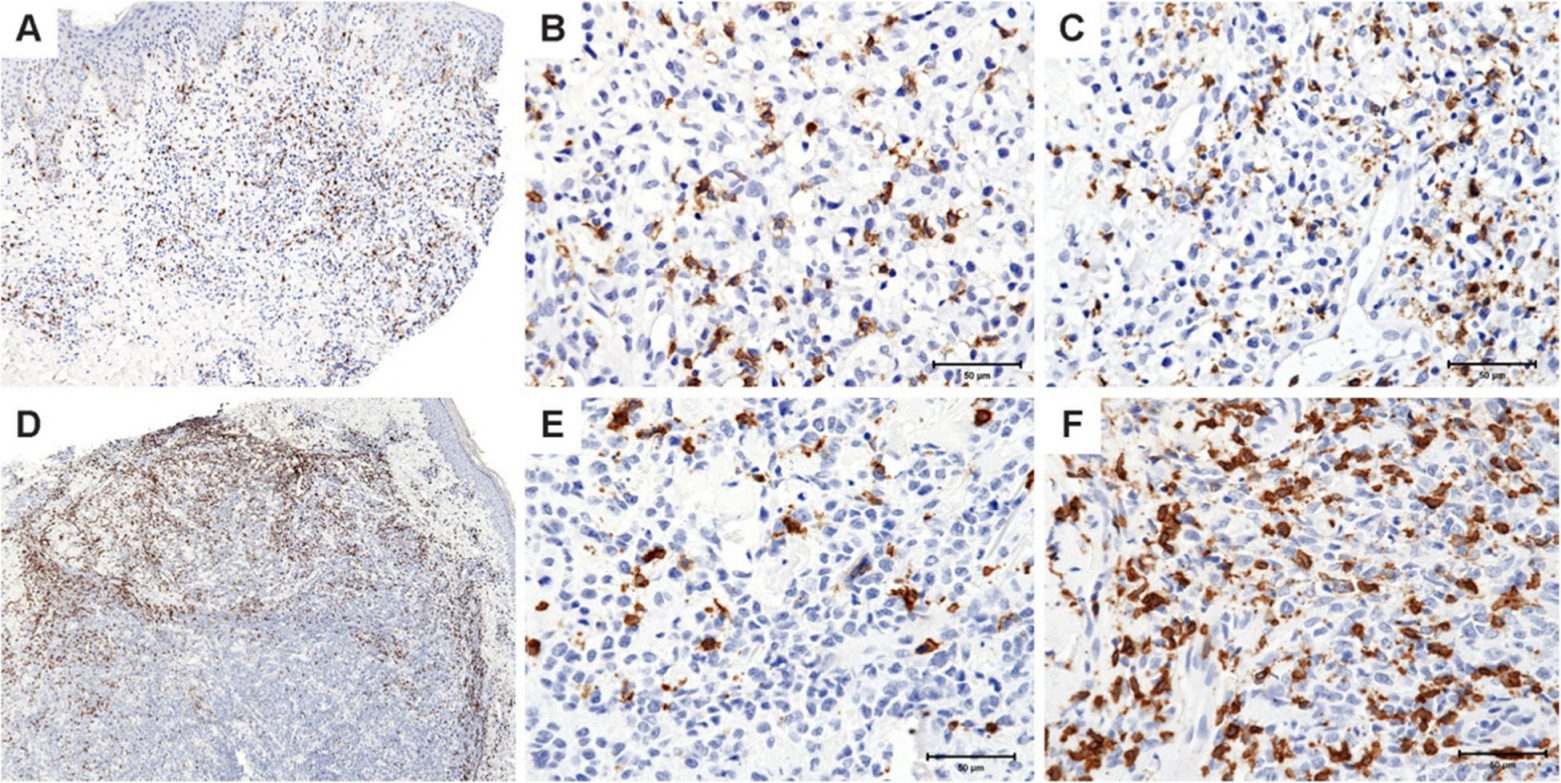
CD8 density in lymphomatoid papulomatosis (LyP) and primary cutaneous anaplastic large cell lymphoma (pcALCL): **a**- LyP showed a regular distribution of CD8 positive cells between center and edge topographies (100X). **b**- Density of CD8 positive cells in the center of LyP (400X). **c**- Density of CD8 positive cells in the edge of LyP (400X). **d**- The density of CD8 is higher in the edge of pcALCL than in the center (100X). **e**- Density of CD8 positive cells in the center of pcALCL (400X). **f**- Density of CD8 positive cells in the edge of pcALCL (400X)

As compared to LyP, pcALCL had a trend toward a higher density of CD8-positive TILs at the edge but not the center of the lesion (*p* = 0.05, Kruskal-Wallis test; Table 2) (Fig. 2). This tendency of enrichment of CD8-positive TILs in the edge as compared to the center of pcALCL group (the CD8 edge/CD8 center ratio) was confirmed to be significantly different between those LyP (1.0 edge to center ratio) and pcALCL groups (2.6 edge to center ratio). The difference in the ratio of edge to center distribution was statistically significant between the two groups (*p* = 0.04, Kruskal-Wallis test; Table 2).

##### CD8-positive tumor infiltrating lymphocytes outnumber FOXP3+ T cells

In the group of CD30+ lymphoproliferative disorders overall, CD8+ TILs significantly outnumbered FOXP3+ cells in both the edges and the centers of lesions (Wilcoxon paired-ranks test, *p* = 0.0014, edge; *p* = 0.014, center). The same was true of LyP and pcALCL lesions taken individually; there was a statistically significant enrichment for CD8 over FOXP3+ cells at the edge of both LyP and pcALCL lesions (*p* = 0.02 and p = 0.02, respectively; Wilcoxon paired-ranks test), although the excess of CD8 did not reach significance at the centers of the lesions (*p* = 0.21 and *p* = 0.12 respectively; Wilcoxon paired-ranks test).

#### Immune inhibitory PD-L1 expression

##### PD-L1 expression is common in both tumor cells and TAMs across categories of cutaneous CD30+ T-cell lymphoproliferative disorders

**Fig. 3:**
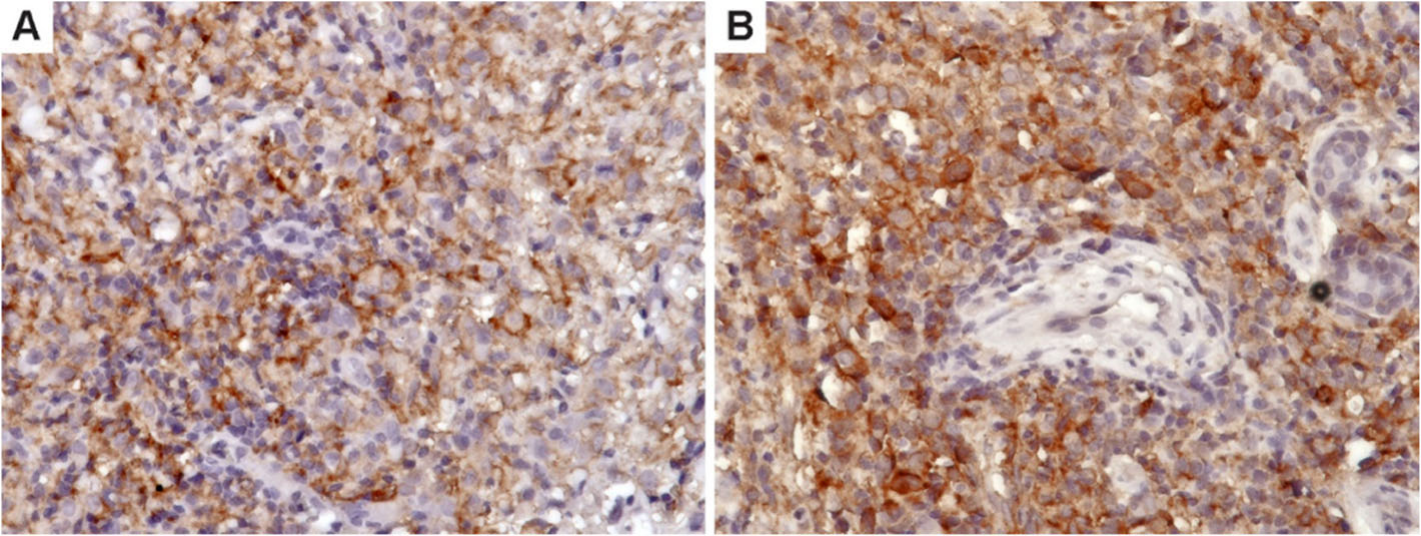
PD-L1 expression in lymphomatoid papulomatosis (**a** – 400X) and primary cutaneous anaplastic large cell lymphoma (**b** – 400X), showing expression in both tumor cells and tissue associated macrophages (TAM)

PD-L1 expression was frequent in pc-CD30-LPD: 50% of LyP cases, 22.2% of pcALCL and 25% of borderline cases expressed moderate to strong intensity PD-L1 in more than 30% of the tumor cells (Fig. 3). There was no difference in PD-L1 expression between LyP and pcALCL groups (*p* = 0.59; Fisher’s exact test; Additional file 1: Table S1). The expression of PD-L1 in the inflammatory immune cell background, mainly in TAMs, was also common and did not differ between the LyP and pcALCL groups (*p* = 0.57; Fisher’s exact test; Additional file 1: Table S1).

##### Correlation among tumor microenvironment, immune inhibitory PD-L1 expression and prognostic factors

We next assessed for associations between features of the tumor microenvironment and clinical features (number of relapses, time until first relapse; Additional file 2: Table S2). There was no statistical correlation between the expression of PD-L1 in the tumor cells or in TAM, CD8/FOXP3 ratio in the center or edge of the lesions, or CD8 edge/center ratio or FOXP3 edge/center ration and time to first relapse. The power of this analysis is quite limited due to the small cohort size.

## Discussion

To better understand the biology of the tumor microenvironment and of providing appropriate immunotherapy as an alternative treatment, many studies have focused on TILs and PD-L1 in tumor tissue [17]. Given the paucity of data about the tumor microenvironment and PD-L1 expression in pc-CD30-LPD, we characterized these parameters and show distinct expression and spatial distribution patterns of Tregs, TILs and PD-L1 expression among pc-CD30-LPD.

De Souza et al. showed that PD-1 TILs were present in both LyP and pc-ALCL tumor microenvironments, but virtually absent in reactive CD30 inflammatory disease [13]. This data supports the relevance of studying the expression of PD-L1 on tumor cells of pc-CD30-LPD, since one of the pathways to down-regulation of effector anti-tumor T-cell activity is by PD-1+ follicular helper T-cells binding to their ligand PD-L1 [13, 17]. In our study, there was no difference in PD-L1 expression on tumor cells or TAM between LyP and pcALCL or borderline groups.

An enlarged pool of FOXP3+ Treg has been demonstrated in different types of solid tumors, where the increased number of Tregs seems to be stage-dependent and correlate inversely with survival rates [18–21]. However, variable findings have been reported in cutaneous T cell lymphoproliferative disorders about the relationship of FOXP3+ Treg density and its relationship with prognosis [13–15]. De Souza et al. reported that the frequency of FOXP3+ Treg was equivalent between LyP and pcALCL, whereas Gjerdrum et al. documented higher concentrations of FOXP3+ Tregs in LyP than in pcALCL [13, 15]. Similar to Gjerdrum et al. study, our findings showed a trend to a higher number of FOXP3-positive cells in LyP compared to the pcALCL group in the center of the lesions, which was confirmed by the FOXP3 edge/FOXP3 center ratio (p-0.05). There was enrichment of FOXP3+ Tregs in the center as compared to the edge of LyP, but not in pcALCL lesions.

Regarding other primary cutaneous T-cell lymphomas, in another study Gjerdrum et al. observed an inverse correlation of FOXP3+ Treg to the tumor stage in cases of MF, with highest median numbers present in cases of plaque or early patch/plaque lesion compared with MF showing tumor or transformation stage [14]. The authors also demonstrated that increasing numbers of FOXP3+ Tregs were associated with improved survival [14]. Moreover, higher numbers of FOXP3+ Treg are related with a better outcome in follicular lymphoma (FL), germinal center-like diffuse large B cell lymphoma (DLBCL) and classical Hodgkin lymphoma (CHL), but have a negative prognostic association in non-germinal center-like DLBCL; and cases of FL with transformation to DLBCL are associated with marked reduction of FOXP3+ Treg in the tumor microenvironment [22, 23]. Among pc-CD30-LPD spectrum of lesions, LyP is self-resolving and was the group for which our study found the highest density of FOXP3 Tregs in the center of the lesions.

Distinct and sometimes opposing roles of FOXP3+ Tregs have been described in the literature: (a) suppressor Tregs, which suppress anti-tumor CD8+ cell-mediated immune responses, similar to the ones found in solid tumors; (b) malignant Tregs in the form of FOXP3+ T-cell lymphomas such as adult T cell leukemia/lymphoma; (c) direct tumor-killing Tregs - some lymphoma cells can be target cells for Tregs suppressive cytotoxicity, suggesting that Tregs can be tumor cell killers; and (d) incompetent Tregs when the number of FOXP3+ Tregs are significantly reduced, representing resting Treg [24]. As a matter of fact, there is evidence that FOXP3+ Tregs are heterogeneous in phenotype and function, consisting of suppressive and non-suppressive subpopulations. Based on the expression levels of FOXP3, CD25 and CD45RA, FOXP3 + CD4+ T-cells can be classified in three subpopulations: (1) naïve or resting Treg cells: FOXP3^lo^CD45RA^+^CD25^lo^ phenotype; (2) effector or activated Treg cells: FOXP3^hi^CD45RA^−^CD25^hi^ phenotype; and (3) non-Treg cells without suppressive activity: FOXP3^lo^CD45RA^−^CD25^lo^ phenotype [8]. Thus the contradictory results in the literature about FOXP3+ Treg may be due to true functional heterogeneity among FOXP3+ cells [8].

Co-inhibitory receptors that regulate effector T cell responses are also associated with induction of exhausted T cells, which is a state of dysfunction that commonly occurs during chronic infections and in the cancer tumor microenvironment due to persistence of antigen. Many studies have been focused on CD8+ T-cells as the prototypic T-cell exhaustion model in the tumor microenvironment [7, 25]. The density of CD8+ TILs has also been shown to correlate with prognosis. Gong Y et al. showed that the number of CD8+ TILs and the degree of PD-L1 expression in lymphoma tissue are both independent prognostic factors in patients with aggressive B cell lymphomas [26]. De Souza et al. demonstrated that CD8+ TILs represent an average of 11% of the infiltrate in LyP cases, and 15% in pcALCL, but they found no significant statistical difference [13]**.** In our study, while there was no significant difference in absolute densities of CD8+ TILs, there was a marked propensity for CD8+ T cells to be denser at the edge than the center of pcALCL but relatively evenly distributed in LyP. Thus while pcALCL and LyP are both infiltrated similarly by CD8+ TILs, in pcALCL they are preferentially enriched at the edge of the tumor. Similarly, FoxP3+ T cells were enriched in the center of LyP lesions but not in pcALCL.

Indeed, the balance of effector T-cells represented by CD8+ TILs and Treg cells in tumors determines the functional outcome of immune responses [27], such that the CD8+/FOXP3+ Treg TILs ratio has been shown to be a better predictor of prognosis and tumor outcome in many tumors [20, 27]. Decreased ratios of tumor-infiltrating CD8+ T-cells to FOXP3+ Treg cells were shown to correlate with poor prognosis [8]. Yang et al. showed that Treg cells in B-cell NHL can attenuate CD8+ TIL function, thereby protecting lymphoma cells from cytotoxic activity [28]. In our study the LyP group had relatively greater central infiltration of both CD8+ TILs (p-0.04) and FOXP3+ T regs (p-0.05) as compared to pcALCL, as if there were greater exclusion of immunoregulatory T cells from pcALCL. The higher greater central enrichment of those immune cells in LyP could be related to the mechanism of spontaneous regression.

## Conclusion

Our findings about the tumor microenvironment in pc-CD30-LPD offer a number of insights for future applications in immunotherapy. The LyP group has a trend of more immune cells throughout the lesion, with higher FOXP3-positive Tregs and CD8-positive TILs in the center than in the edge compared with the pcALCL group. PD-L1 expression and CD8+/FOXP3 Treg TILs ratios in tumor tissue have been correlated with prognosis across cancer types [20, 29], giving support to a holistic approach to determining tumor microenvironment status as a prognostic factor. Our study is limited by a relatively small sample size; nevertheless it adds important new data about the immune microenvironment of CD30+ cutaneous lymphoproliferative disorders that may be amenable to manipulation via immunotherapy.

## Supplementary information

**Additional file 1:** **Table S1.** PD-L1 expression in tumor cells and Tumor Associated Macrophages (TAMs) of LyP and pc-ALCL groups

**Additional file 2:** **Table S2.** Correlation among PD-L1 staining, distribution of TILs, and time until relapse (Cox proportional hazards regression)

## Data Availability

The data and materials used in this current study are available from the corresponding author on reasonable request.

## Abbreviations

CHL: Classical Hodgkin lymphoma
DLBCL: Diffuse large B cell lymphoma
EBER: EBV-associated small RNAs
FL: Follicular lymphoma
HPF: High-power fields
ISH: In situ hybridization
LyP: Lymphomatoid papulomatosis
MF: Mycosis fungoides
pcALCL: primary cutaneous anaplastic large cell lymphoma
pc-CD30-LPD: Primary cutaneous CD30+ lymphoproliferative disorders
PD-L1: Programmed death ligand 1
SPSS: Statistical Package for Social Sciences
TAM: Tissue associated macrophages
TILs: Tumor infiltrating lymphocytes
WHO – EORTC: World Health Organization – European Organization for Research and Treatment of Cancer
WHO: World Health Organization
