# Supplementary material for: Clinicopathologic and microenvironmental analysis of primary cutaneous CD30-positive lymphoproliferative disorders: a 26 year experience from an academic medical center in Brazil
Source: Diagn Pathol. 2019 Oct 22;14:115. doi: 10.1186/s13000-019-0900-7 (PMC6805531; doi:10.1186/s13000-019-0900-7)
Supplement: Supplementary file 1 — Additional file 1: Table S1. PD-L1 expression in tumor cells and Tumor Associated Macrophages (TAMs) of LyP and pc-ALCL groups [file 13000_2019_900_MOESM1_ESM.docx]

**Table S1. PD-L1 expression in tumor cells and Tumor Associated Macrophages (TAMs) of LyP and pc-ALCL groups**

|  | PD-L1 NEGATIVE  % (n) | PD-L1WEAK  % (n) | PD-L1 STRONG  % (n) | LyP vs c-ALCL |
| --- | --- | --- | --- | --- |
| LyP Tumor cells | 38% (3) | 12% (1) | 50% (4) | p = 0.59 |
| pc-ALCL Tumor cells | 56% (5) | 22% (2) | 22% (2) |  |
| LyP TAMs | 25% (2) | 38% (3) | 38% (3) | p = 0.57 |
| pc-ALCL TAMs | 33% (3) | 11% (1) | 56% (5) |  |

Fisher’s exact test
