# Supplementary material for: Clinicopathologic and microenvironmental analysis of primary cutaneous CD30-positive lymphoproliferative disorders: a 26 year experience from an academic medical center in Brazil
Source: Diagn Pathol. 2019 Oct 22;14:115. doi: 10.1186/s13000-019-0900-7 (PMC6805531; doi:10.1186/s13000-019-0900-7)
Supplement: Supplementary file 2 — Additional file 2: Table S2. Correlation among PD-L1 staining, distribution of TILs, and time until relapse (Cox proportional hazards regression) [file 13000_2019_900_MOESM2_ESM.docx]

**Table S2. Correlation among PD-L1 staining, distribution of TILs, and time until relapse (Cox proportional hazards regression)**

|  | n | Time until first relapse |
| --- | --- | --- |
| Tumor cells PD-L1+ | 10 | chi2 = 1.59, p=0.21 |
| TAMs PD-L1+ | 10 | chi2 = 0.65, p=0.42 |
| CD8/FoxP3 ratio (tumor center) | 10 | chi2 = 0.38, p = 0.53 |
| CD8/FoxP3 ratio (tumor edge) | 10 | chi2 = 0.32, p = 0.56 |
| Edge/center ratio of FoxP3 | 10 | chi2 = 0.13, p = 0.72 |
| Edge/center ratio of CD8 TILs | 10 | chi2 = 0.0 , p = 0.99 |
